# Nurses’ knowledge of blood transfusion in medical training centers of Shahrekord University of Medical Science in 2004

**Published:** 2010

**Authors:** Yosef Aslani, Shahram Etemadyfar, Kobra Noryan

**Affiliations:** * Department of Medical Surgical Nursing, Shahrekord University of Medical Sciences, Shahrekord, Shahrekord, Iran

**Keywords:** Knowledge, blood transfusion, nurse, blood components

## Abstract

**BACKGROUND:**

Using blood and blood components is a common therapeutic procedure in hospitals. Nurses have an important role in a safe blood transfusion. Therefore, it is crucial for nurses to have sufficient knowledge of situations, amount and methods of using blood components, possible side effects and necessary cares. This study investigated nurses’ knowledge of blood transfusion.

**METHODS:**

This was a cross-sectional descriptive study on 117 nurses in medical training hospitals of Shahrekord University of Medical Sciences in 2004, aiming to evaluate their knowledge of blood transfusion. Data were collected using a questionnaire including 4 sections and 29 questions. Sections included demographic data, nurses’ knowledge of blood components, nurses’ knowledge of blood components infusion techniques, and nurses’ knowledge of indication and side effects of blood components infusion. Knowledge scores were first coded and then categorized in three levels of good, average, and poor. Data were analyzed using SPSS software.

**RESULTS:**

The nurses’ knowledge of blood and blood component, techniques of blood components infusion, and its indication and side effects was average (66.7%, 65.8% and 59%, respectively).

**CONCLUSIONS:**

The findings showed that the nurses’ knowledge of blood and blood component was average and insufficient. Therefore, it is recommended to activate the blood transfusion committees in hospitals to increase the quality of this common procedure and prevent side effects by in-service trainings of nurses.

Blood is very valuable especially in saving lives of patients. Blood components are expensive and their preparation is limited. Therefore, they should be correctly selected and used for patients by all means.^1^ The aim of blood and blood components transfusion in medical treatments is to provide suitable and safe blood products to achieve best clinical outcomes.^2^ In spite of its vital role in saving lives, blood transfusion is associated with risks.^3^ Making mistakes in blood transfusion and insufficient control of patients who receive blood during the transfusion are among causes of death for such patients.^4^ Since there is no substituting product for human blood, the need for blood transfusion is still continuing.^5^ More than 50% of patients hospitalized in intensive care units and 50% to 70% of patients in surgical and orthopedic wards need blood transfusion.^6^

Annual reports in Britain show serious risks of blood transfusion such as neglect in identification of blood type and its components, wrong identification of patients, and neglect in controlling patients during transfusion as main causes of mistakes.^7^ Therefore, considering the severe need for blood and blood components, along with limited sources and limited possibility of preparing each blood product, it is crucial to try by all means to increase the knowledge of medical personnel and providing necessary education to reduce consumption of complete blood and to use just the components necessary for patients’ health, in order to reduce blood waste and transfusion complications.^1^ Studies showed that nurses and nursing students do not have sufficient information and correct performance suitable to the importance of the issue.^8^ Nurses play a significant role in correct, scientific and safe usage of blood and its components and if they can do it correctly, the probability of incidence of blood transfusion risks will be reduced to minimum.^9^

This study aimed to assess nurses’ knowledge of blood components transfusion in medical training hospitals of Shahrekord University of Medical Sciences. The detailed objectives of the study included determining demographic data of the sample, nurses’ knowledge of blood components, and its transfusion techniques, nurses’ knowledge of usage and complications of blood components transfusion.

## Methods

This was a cross-sectional descriptive study on 117 nurses working in medical training hospitals of Kashani and Hajar in Shahrekord. Sampling was simple and purposive. Data were collected using a questionnaire including 4 sections and 29 questions: demographic data, nurses’ knowledge of blood components (10 questions), nurses’ knowledge of blood components infusion techniques (10 questions), and nurses’ knowledge of indication and side effects of blood components infusion (9 questions). The content validity was used to check the validity of the questionnaire and the reliability was checked by test retest and the correlation coefficient was 0.92.

The researcher went to the hospitals and distributed the questionnaires among the subjects, explained the objectives of the study to them and gave them sufficient time to complete the questionnaire and then, collected the completed ones. Knowledge scores were first coded and then, categorized in three levels of good, average and poor for detailed objectives ranked from 2 to 4. Data were analyzed using descriptive (frequency distribution and mean) and inferential (chi-square) statistical methods, based on the study objectives.

The ethical committee of Shahrekord University of Medical Sciences approved the study.

## Results

Findings showed that 37.2% of subjects were aged between 23-25 years, 31.4% were 26-33 year-old and 31.4% were 34-56 year-old. Regarding career history, 43.7% had 1-3 years of work experience, 28.7% had 4-10 years and 27.6% had 11-28 years. Also, 83% of subjects were women and 17% were men; 38.7% were single and 85.2% were married. The educational degree of 7.8% was health diploma, 6.9% had a college degree and 85.2% had bachelor of nursing. The employment was permanent contract for 56.3%, internship for 33.9% and temporal contract for 9.7%.

Moreover, the results of the study regarding the nurses’ knowledge of blood and its components showed that 95.7% of the nurses in the study answered correctly to the following question: “What kind of blood sample of patients should be sent to blood bank to do the crossmatch”. The lowest frequent correct answer was to the question “When would be the appropriate transfusion time after receiving the blood and its components from blood bank”; 81.2% answered wrongly to this question. Also, 21.4% of subjects had a good level of knowledge, 66.7% had average and 12% had poor level of knowledge. Also, the results of study about nurses’ knowledge of blood transfusion techniques showed that the second frequent question answered correctly by most subjects was on routine cares during transfusion (81.2%) and the lowest frequent correct answer was to the question on routine cares after blood transfusion which 93.2% answered it wrongly. Furthermore, the results of the study about nurses’ knowledge of indication and complications of blood transfusion showed that the highest level of knowledge was related to the question “Which of the blood components will be used in anemia treatment”; 93.2% answered it correctly. The lowest level of knowledge was related to the question “What is the best component to treat the shortage of coagulation factors” which 61.8% answered it wrongly. Also, 16.2% of subjects had good level of knowledge, 59% had average and 24.8% had poor level of knowledge. Therefore, it can be concluded that nurses’ knowledge of indication and complications of blood transfusion was average.

## Discussion

The results showed that nurses’ knowledge of blood and its components was average. In a study in Turkey, most nurses had a score of 50 out of 100 for knowledge and performance which means average score.^5^ However, in the present study, 36.8% of nurses got a score of 18.33 out of 29 in knowledge and information, which was higher than the average. In France, nurses’ knowledge and performance in this field was reported weak and the lowest level of knowledge was on the time of blood transfusion that was related to lack of identifying patients and identifying the required components in 54% of cases.^9^

Also, we found that nurses’ knowledge of blood transfusion techniques is average. Teymouri et al showed that nurses’ knowledge and performance of using needles with appropriate diameter was good but they did not have correct and scientific knowledge of indications and method of heating blood.^10^ In the study of Tabiei et al, just 26 nurses (25%) had the knowledge of beginning blood transfusion half an hour after the blood is delivered.^11^ A study in Turkey related to the same issue reported 17.2% statistic.^5^ In addition, nurses’ knowledge of indication and complications of blood transfusion was average.

The results of the study showed that the average knowledge of nurses can increase probable incidence of risks related to blood transfusion and reduce the quality of health care. Therefore, researchers recommend activation of a blood transfusion committee in hospitals to control reports of blood transfusion and its components as well as possible complications in wards, and to develop and execute inservice training programs for personnel emphasizing the weak points to increase their information and knowledge and continuously supervise this task.

The authors declare no conflict of interest in this study.
